# Platelet-Rich Plasma in Female Androgenic Alopecia: A Comprehensive Systematic Review and Meta-Analysis

**DOI:** 10.3389/fphar.2021.642980

**Published:** 2021-05-06

**Authors:** Shuying Zhou, Fei Qi, Yue Gong, Chenxi Zhang, Siqi Zhao, Xutong Yang, Yanling He

**Affiliations:** ^1^ Department of Dermatology, The 305 Hospital of PLA, Xicheng, China; ^2^ Capital Medical University, Beijing Chaoyang Hospital, Beijing, China

**Keywords:** platelet-rich plasma, female androgenic alopecia, female pattern hair loss, systematic review, meta-analysis

## Abstract

**Introduction:** The population of young women who suffered from female pattern hair loss (FPHL) or female androgenic alopecia (AGA) is gradually increasing. Platelet-rich plasma is a novel and promising therapeutic method as a nonsurgical treatment for FPHL.

**Objective**: To summarize different preparation methods of PRP and treatment regimes in FPHL, qualitatively evaluate the current observations, and quantitively analyze the efficacy of PRP in FPHL treatment.

**Methods:** Six databases, MEDLINE, EMBASE, Web of Science, Cochrane Central Register of Controlled Trials, LILACS, and CNKI, were searched with terms “platelet-rich plasma,” synonyms for AGA and FPHL. Meta-analysis was conducted with enrolled observational studies and randomized controlled trials separately.

**Results:** We evaluated 636 studies and 12 trials from all searched databases. A total of 42 studies of 1,569 cases, including 776 female participants covering 16 randomized controlled trials and 26 observational trials, were included for qualitative synthesis study and systematic review. PRP showed positive efficacy in treating FPHL in hair density compared to the control groups with odds ratio (OR) 1.61, 95% CI 0.52–2.70, and compared to baseline with OR 1.11, 95% CI 0.86–1.37.

**Conclusion:** PRP showed excellent efficiency as a novel therapy of FPHL through hair density evaluation. Further studies are needed to define standardized protocols, and large-scale randomized trials still need to be conducted to confirm its efficacy.

## Introduction

Female pattern hair loss (FPHL), with an alternative name female androgenic alopecia (AGA), is the most common type of hair loss affecting 6.0% of Chinese women, among which 3.6% were under 40-year-old (Wang et al., 2010). FPHL is characterized by progressive follicular miniaturization and the accompanying conversion of terminal follicles into vellus-like follicles (Trueb, 2002). Patients may present with hair density reduction, hair thinning, and widening of the area especially on the center of the scalp (Olsen, 2001). Those changes may lead to serious psychological impacts (van Zuuren et al., 2012) on one’s self-esteem, interpersonal relationships, and the social status (Cash, 2001). Although multiple nonsurgical therapeutic methods like topical minoxidil, oral finasteride, and low-level laser comb had been introduced to FPHL treatment, more large-scale–randomized controlled trials still need to be investigated to confirm their efficacy (van Zuuren et al., 2016). Despite decreased number of more actively proliferating progenitor cells, the current studies have presented an unaltered number of hair follicle stem cells in a hair loss scalp (Garza et al., 2011). Hence the application of autologous stem cells including autologous micro-grafts enriched of human follicle cells (HF-MSCs) as well as platelet-rich plasma has been explored and gradually been introduced to clinical use (Gentile et al., 2019).

Platelet-rich plasma (PRP) is a preparation of an enriched platelet autologous plasma in which the concentration of platelet is above normal contained in the whole blood (Wu et al., 2016). Platelets are able to secrete growth factors, adhesion molecules, and chemokines. After being activated, those effective factors interact with the local environment and promote cell differentiation, proliferation, and regeneration (Weibrich et al., 2002; Davì and Patrono, 2007; Sánchez-González et al., 2012). Published data also highlighted that PRP contains major growth factors, including basic fibroblast growth factor (bFCF), platelet-derived growth factor (PDGF), vascular endothelial growth factor (VEGF), epidermal growth factor (EGF), transforming growth factor-*β* (TGF-*β*), and insulin-like growth factor-1 (IGF-1) (Cervelli et al., 2012). In the past decade, PRP has been studied and widely used in alopecia, acne scarring, skin rejuvenation, chronic wounds, and vitiligo (Hesseler and Shyam, 2019). Comparing with traditional nonsurgical therapies and the surgical approaches such as hair transplantation, PRP is believed as a promising treatment of AGA with lower cost and fewer adverse effects. However, only a limited number of publications focus on the utilization of PRP in AGA treatment, and few studies or reviews about the application of PRP in FPHL have been performed.

In this study, we summarized different PRP preparation methods, reviewed the various treatment regime in FPHL that are reported in the previous studies, qualitatively evaluated the current observations, and quantitively analyzed the efficacy of PRP in FPHL treatment. This work may offer a reference to the clinical workers and associated FPHL patients.

## Methods

This meta-analysis was conducted in accordance with the Preferred Reporting Items for Systematic Reviews and Meta-Analysis (http://www.prisma-statement.org) and Meta-analysis Of Observation Studies in Epidemiology (MOOSE) statement (Stroup et al., 2000).

### Literature Screening

Two investigators independently conducted a systematic search of studies published before October 29, 2020, using the databases MEDLINE via PubMed, embase, Web of Science via Ovid, Cochrane Central Register of Controlled Trials (CENTRAL), LILACS, and CNKI. The strategy of search terms was composed of at least one term from two search blocks: the term “platelet-rich plasma” and synonyms for androgenic alopecia (AGA) and female pattern hair loss (FPHL). All synonyms were found based on MESH and ENTRÉE (Supplementary Material S1).

### Study Selection

Original studies include observational studies (i.e., case series, cross-sectional, case-control, and cohort) and randomized trials of platelet-rich plasma (PRP), and AGA in woman patients in English, German, Swedish, Norwegian, Spanish, Danish, Turkish, and Chinese were all eligible for inclusion. Exclusion criteria were reviews, studies only included male patients, abstracts, unpublished studies, and lack of raw data. Conference reports were also excluded for insufficient details for analysis. Two reviewers screened the titles and abstracts of the identified studies. If the information provided in the abstracts was not sufficient to access the eligibility, a full-text evaluation was conducted. All observational studies and randomized trials will be recruited for qualitative analysis, and only randomized controlled trials will be analyzed quantitively and enrolled in the meta-analysis in this work. Two authors also evaluated the quality of the included studies independently. Any disagreement was resolved through discussion or decided by a third person.

### Data Extraction

Two reviewers completed the data extraction work independently with the following information: author, year of publication, journal name, country, methods of PRP preparation, treatment regime, and the number of cases/controls (total, exposed, nonexposed). In case of disagreement, a third person conducted a further assessment.

### Endpoint Definition

The efficacy of PRP was evaluated by an increase in hair density, increment of hair count, improvement in the hair-pull test, satisfaction of patients from the questionaries, and changes of hair thickness compared with photos taken before and after the treatment sections. Given that various test methods were taken through the studies we included, only the most widely used methods would be set at the endpoints for all-pooled studies.

To access the safety of PRP, all-side effects, including local injection pain, headache, increasing scalp sensitivity, and any allergic effects, would be recorded.

### Data Analysis

We conducted the meta-analysis with Stata statistical software 15.0 (Metrika Consulting, Stockholm, Sweden) using the “metan” command. A random-effect analysis with the method of Dorsmanin and Laird (Higgins et al., 2020) was taken, given the considerable heterogeneity. We chose 95% confidence intervals (CI) and I (Trueb, 2002) to access statistical heterogeneity (Higgins et al., 2003; Cook and Reed, 2015): 30–60% as moderate heterogeneity, 50–90% substantial heterogeneity, greater than 75% considerable heterogeneity (Higgins et al., 2020). Funnel plots and Egger tests were performed for publication bias assessment with asymmetry in the funnel plot and a *p*-value that was less than 0.1. Eggers’s test would overrule visual inspection if results diverged between the two methods above.

Clinical heterogeneity was measured by stratification, for example, subgroup analysis of the study types, i.e., randomized controlled trials and observational trials. The observational trials were further stratified into case series, cohort studies, cross-sessional studies, and case-control studies. We also conducted a subgroup analysis based on different endpoint definitions, e.g., hair density, hair count, and increment in hair density. Subgroup analysis with different PRP preparation methods and PRP concentrations were only performed with sufficient data.

The quality of each study was evaluated based on Joanna Briggs Institute Critical Appraisal tools for case-series studies (Munn et al., 2020), Newcastle-Ottawa Scale (Stang, 2010) for cohort studies and case-control studies, and Cochrane Risk of Bias Tool for Randomized Controlled Trials (Higgins et al., 2011). Three groups of study quality were defined: high, middle, and low. Rovis was used for the presentation of the quality of each study (McGuinness and Higgins, 2020).

## Results

### Literature Search

**FIGURE 1:**
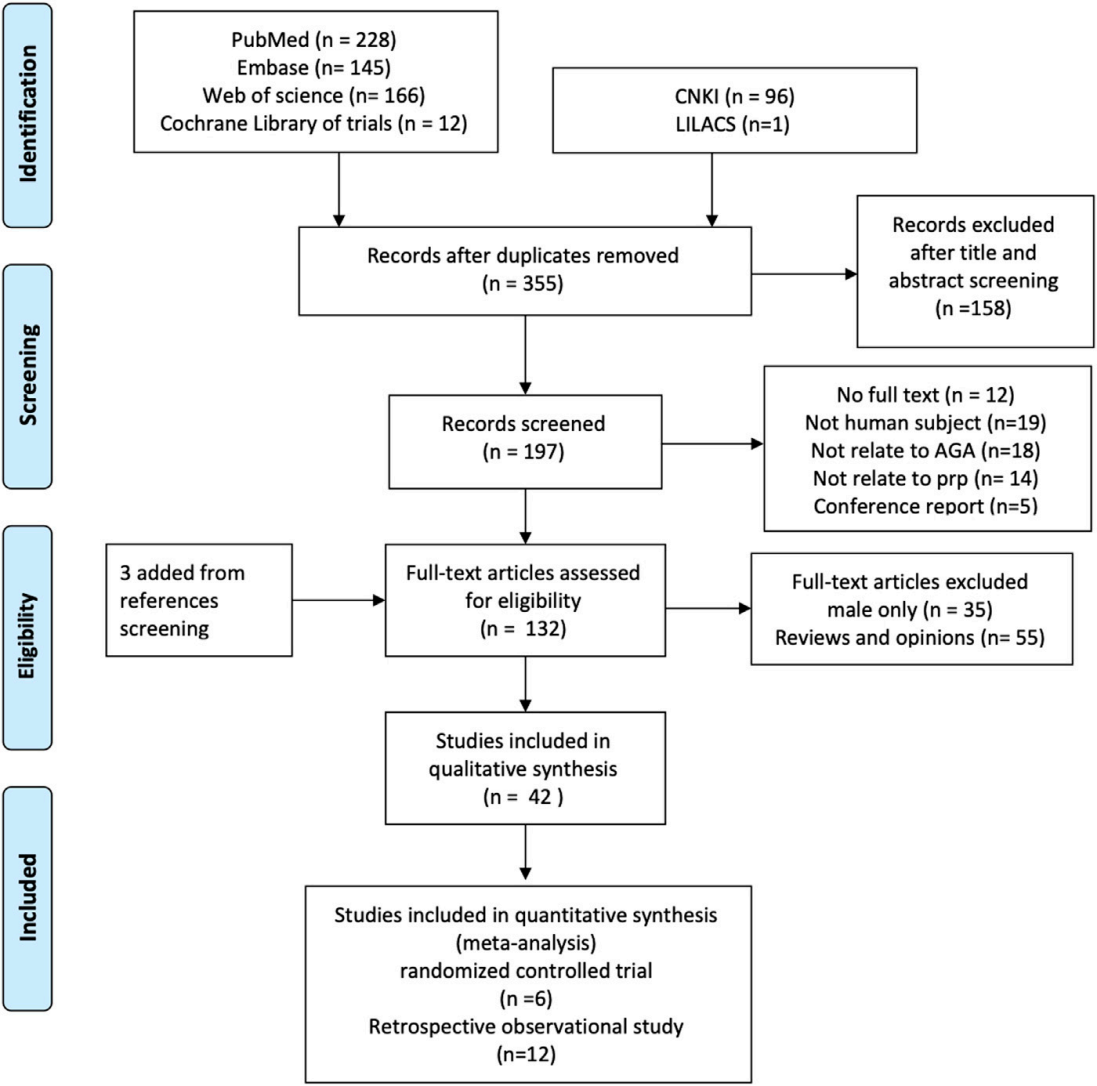
The process of literature screening.

We found 636 literature and 12 trials from databases and three additional references by reference screening. Six hundred and three studies were excluded for reasons including duplicated, male patients only, and not in human subjects (Figure 1).

Although only the PRP effect on a female would be evaluated in this study and given only a few pure women studies were found, studies involved in both female and male patients would also be pooled in this work. A total of 42 studies, 1,569 cases including 776 female participants covering 16 randomized controlled trials and 26 observational trials, were included for qualitative synthesis study and systematic review.

### Study Characters

**FIGURE 2:**
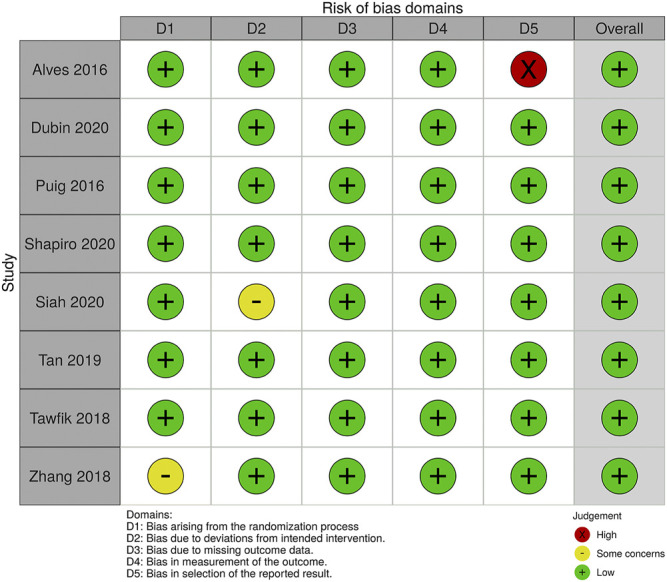
The Risk of Bias evaluation of randomized trials.

**FIGURE 3:**
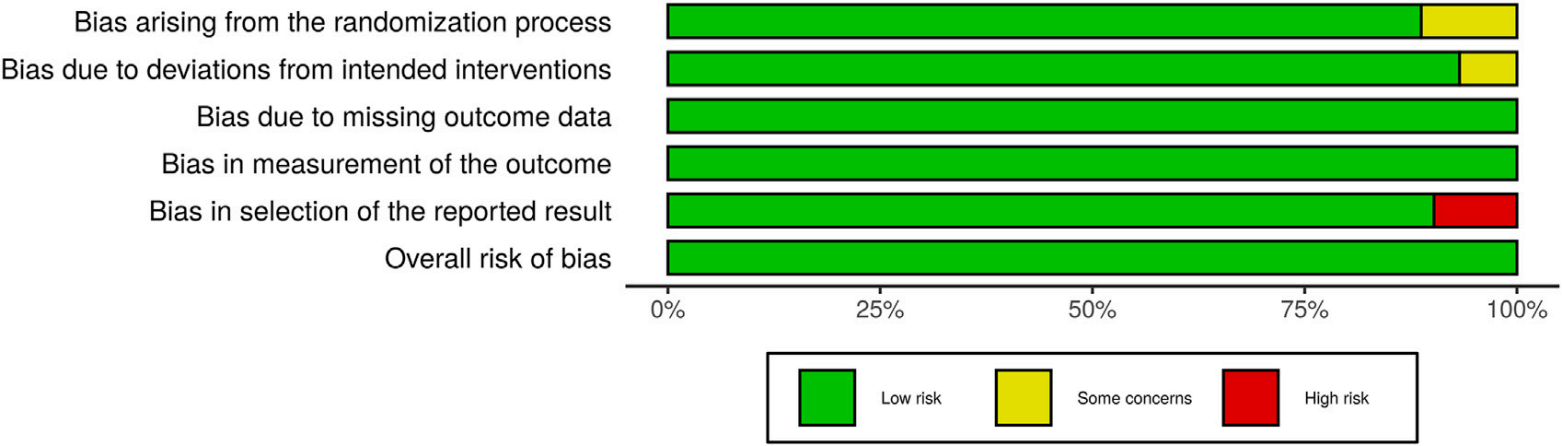
Summary of Risk of Bias in Randomized Controlled Trials.

Studies with various group settings were enrolled (Table 1). Eight out of 16 randomized controlled trials investigated the effect of PRP comparing with placebo, in which 6 of which were done in half-headed comparation. Four studies evaluated the differences of the effect of PRP combination therapy with topical minoxidil, oral finasteride, laser, and polydeoxyribonucleotide (PDRN), while the other four accessed the different efficacy from treating strategy, i.e., PRP concentration, centrifugation protocol, treatment sessions, and treatment doses. The risk of bias of all-eight–randomized placebo-controlled trials was graded by two reviewers (Figures 2, 3).

**TABLE 1:** The study characters.

Author/year	Country	Trial characters	Subject characters	Objectives
Abaroa 2016	Mexico	RCT	10 males and 6 females	Efficacy of APRP
Alves 2016	Spain	RCT	23 males and 12 females	Efficacy of APRP with half-headed
Alves 2018	Spain	RCT	13 males and 11 females	Efficacy of APRP combines with topical minoxidil and oral finasteride, half-headed
Anitua 2017	Spain	UCT	13 males and 6 females	Efficacy of APRP
Bruce 2019	USA	RCT	20 females	Efficacy of APRP with half-headed
Butt G 2020	Pakistan	RCT	17 males and 5 females	Efficacy of APRP with SVF-PRP
Butt G 2019	Pakistan	UCT	20 males and 10 females	Efficacy of APRP with minoxidil
Dina 2019	USA	Case report	2 females	Efficacy of APRP
Dubin 2020	USA	RCT	30 females	Efficacy of APRP with placebo
El-husseiny 2020	Egypt	UCT	13 males and 10 females	Efficacy of APRP in double-spin and single-spin, half-headed
Garg 2017	India	UCT	65 males and 50 females	Efficacy of APRP
Gentile a 2020	Italy	UCT	15 females	Efficacy of APRP combined with microneedling technique, low-level laser therapy
Gentile b 2020	Italy	UCT	63 males and 27 females	Efficacy of APRP activated APRP compared with nonactivated PRP
Gentile 2018	Italy	UCT	18 males and 5 females	Efficacy of APRP
Gkini 2014	Greece	UCT	20 males and 2 females	Efficacy of APRP
Hausauer 2018	USA	RCT	29 males and 10 females	Efficacy of APRP with half-headed
Ho 2020	USA	UCT	24 females	Efficacy of APRP with minoxidil
Juhasz 2020	USA	UCT	74 females and 30 males	Efficacy of APRP
Kang 2014	South Korea	RCT	15 males and 11 females	Efficacy of APRP with placebo
Laird 2018	Spain	Patients survey	41 females	Efficacy of APRP
Lee 2015	South Korea	RCT	40 females	Efficacy of APRP plus PDNR and PDNR monotherapy
Makki 2020	Egypt	UCT	13 males and 37 females	Efficacy of APRP
Paththinige 2018	Siri lanka	UCT	27 males and 1 females	Efficacy of APRP
Puig 2016	USA	RCT	16 females	Efficacy of APRP with placebo
Qu 2019	China	UCT	51 males and 37 females	Efficacy of APRP
Rossano 2017	Italy	UCT	25 males and 16 females	Efficacy of APRP
Schiavone 2018	Italy	UCT	102 males and 66 females	Efficacy of APRP
Schiavone 2014	Italy	UCT	42 males and 22 females	Efficacy of APRP
Sclafani 2014	USA	UCT	9 males and 6 females	Efficacy of APRP
Shapiro 2020	USA	RCT	18 males and 17 females	Efficacy of APRP with half-headed
Siah 2020	United Kingdom	RCT	1 males and 9 females	Efficacy of APRP
Singhal 2015	India	UCT	1 males and 2 females	Efficacy of APRP
Starace 2019	Italy	UCT	10 females	Efficacy of APRP
Takikawa 2011	Japan	RCT	16 males and 10 females	Efficacy of APRP with D/P MPS
Tan 2019	Singapore	RCT	33 males and 22 females	Efficacy of APRP with half-headed
Tawfik 2018	Egypt	RCT	13 females	Efficacy of APRP with placebo
Zolfaghari 2020	Iran	UCT	4 males and 9 females	Efficacy of APRP
Zhang 2018	China	RCT	28 males and 32 females	Efficacy of APRP with placebo
Navarro 2015	Spain	UCT	50 males and 50 females	Efficacy of APRP
DeVasconcelos 2015	Brazil	UCT	9 males and 7 women	Efficacy of APRP
Yang 2020	China	RCT	27 males and 5 females	Efficacy of APRP with minoxidil
Zhang 2020	China	RCT	36 males and 34 females	Efficacy of APRP with lacer and placebo

RCT, randomized controlled trials; UCT, uncontrolled clinical trials; APRP, autogenous platelet-rich plasma; SVF, stromal vascular fraction; PDNR, polydeoxyribonucleotide; D/P MPs, dalteparin and prota-mine microparticles; USA, the United States of America.

**FIGURE 4:**
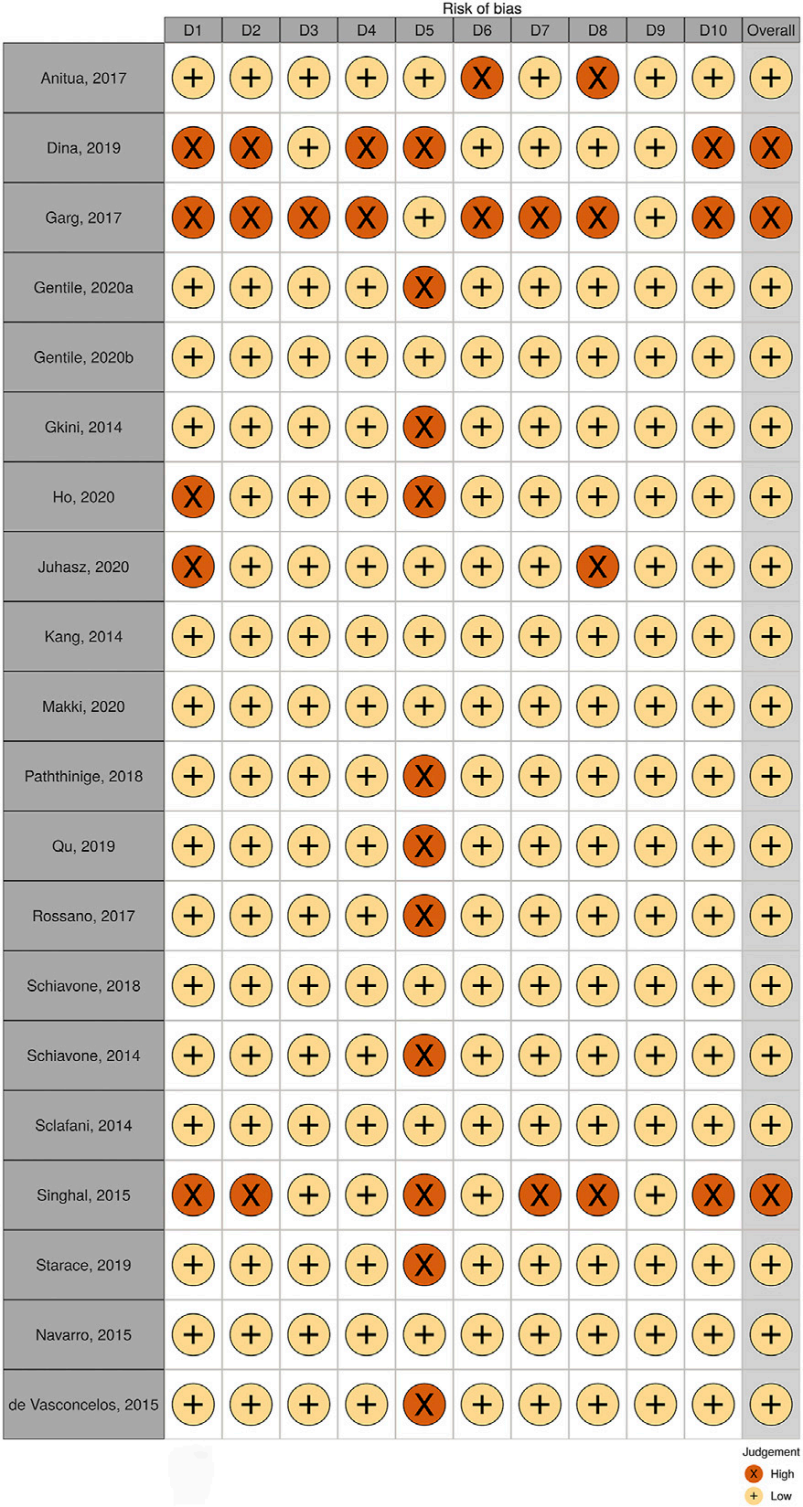
Risks of Bias evaluation of case series.

Observation studies including 21 case series, 2 case reports, one patient survey, one treatment protocol, and one treatment experience were enrolled, while one case series containing only one female patient was abandoned. Quality of the total 20 case series was accessed, three of which were excluded for high risk after evaluation (Figure 4).

### Study Subjects

Among all 42 studies, we only found seven studies that recruited only female participants. The mean age of the total-enrolled patients was above 18 years old and between 25 and 35 years old. Most female patients had a history of AGA for at least 3–5 years with type I to III FPHL on Ludwig scale (Harrison, 2017) or 1–5 on Sinclair score system (Kasprzak et al., 2019). Patients who had previous hair transplantation, a drug-taking history that could cause hair loss or any inflammations, scars, or erythema on the scale were excluded. Laboratory tests, including hemoglobin, platelet count, serum ferritin, liver function, thyroid function, and female hormone profile, were checked for excluding any other autoimmune or systematic diseases that could cause hair loss. Acute or chronic infections should also be tested before using PRP.

### Platelet-Rich Plasma Preparation

The methods of PRP preparation vary in studies (Table 2). PRP kits were commonly used from a different company and centrifugation protocol. The choices of activators and anticoagulation vary depending on PRP kits and study purposes. In all-enrolled studies, calcium gluconate in a 10:1 ratio and sodium citrate were mainly added as activators and anticoagulants, respectively. Moreover, platelet concentration differs from 1.5 times to seven-fold as whole blood according to the PRP preparation protocols.

**TABLE 2:** Preparation of PRP in studies.

Author/year	Blood drawn	Centrifugation protocol	Activators	Anticoagulant	Platelet concentration
Abaroa 2016	20–30 ml	Double-spin method, 20–30 ml of cititrated blood, centrifuged at 1,800 rpm for 10 min, abandon buffy coat, 3,000 rpm for 10 min	Calcium gluconate in a 10:1 ratio	6.8% sodium citrate	200–400% over basal blood
Alves 2016	18 ml	460 g for 8 min	0.15 ml 10% calcium chloride immediately before application	2 ml of 3.8% sodium citrate	Approcimately 3 times higher than whole blood
Alves 2018	18 ml	460 g for 8 min	PRP kits	2 ml of 3.8% sodium citrate	Approcimately 3 times higher than whole blood
Anitua 2017	18 ml	580 g for 8 min	PRGF activbitor	9 ml tubes 3.8% sodium citrate	
Bruce 2019	60 ml	Dual spin centrifugation, 1,500 rpm for 10 min, removal of red cell layer, additional centrifugation at 3,500 rpm for 10 min, G force 684, and radius of 50		8 ml citrate dextrose solution A	
Butt G 2020	9 ml	650 g for 10 min		PRP kit (tray life tube gel) comprising a mixture of polymers that sepatated plasma and sodium citrate	
Butt G 2019	9 ml	1,000 rpm for 10 min		PRP kit (tray life tube gel) comprising a mixture of polymers that sepatated plasma and sodium citrate	
Dina 2019	9 ml	1,100 g for 6 min	0.5 m calcium chloride	Sodium citrate	
Dubin 2020	22 ml	Eclipse PRP system 3500 revolutions per minute for 10 min			
El-husseiny 2020	10 ml	Double-spin: 800D at 1,12 g (100 rpm) for 10 min, plasma for 448 g units (2,000 rpm) for 10 min, single-spin: 1,372 g units (3,500 rpm) for 10 min	1 ml calcium gluconate (1:9)	Tri-sodium citrate	
Garg 2017	13.5 ml	REMI [centrifuged for 4 min at 3,000 rpm (revolutions per minute)]	Y cell bio kit	1.5 ml of ACD-A solution	It gives 5–7 times the concentration of the baseline platelet count
Gentile a 2020	55 ml		Three different kit: C-Punt, i-stem, MAG-18		
Gentile b 2020	55 ml or 18 ml for APRP, ANAPRP for 18 ml	A-PRP:C-PunT prepration system, 1,200 rpm per 10 min, followed 1,200 rpm for 5 min, MAG-18 PRP kit, 3,000 rpm for 6 min, then 3,000 rpm for 2 min, AA-PRP: Cascade-selphyl, 1,100 g per 10 min	Calcium gluconate in a 10:1 ratio	Sodium citrate (ACD)	
Gkini 2014	16 ml	RegenKit-BCT-3, 1,500 g for 5 min	Calcium gluconate	Dodium citrate solution	5.8 times as whole blood
Hausauer 2018	22 ml	3,500 revolutions per minute for 10 min	EclipsePRP kits		4 to 6 times the platelet concentration of whole blood
Ho 2020	8 ml	1,500 g 5 min	RegenKit-BCT		1.5 times, total 5 ml
Juhasz 2020	8 or 16 ml	1,500 g 5 min	regenkit-BCT		1.6 times, total 5 ml
Kang 2014	60 ml		SmartPReP	Sodium citrate solution	
Laird 2018	8 ml	Double-spin cycle	20 mg of acell MatriStem micro matrix + 1cc of 10% calcium gluconate	Dextrose solution A	
Lee 2015	60 ml		SmartPReP	4% sodium citrate solution	
Makki 2020	10 ml	400 g for 10 min, upper part for 800 g 10 min			
Paththinige 2018	18 ml	Male 3,000 rpm 4 min, female 3,000 rpm for 3 min; fastened PRP kit was for 3,200 rpm, 6 min	Calcium gluconate in a 10:1 ratio	Sodium citrate	
Puig 2016	60 ml				2.75 to 3.4 times
Qu 2019	40 ml	3,300 rpm for 4 min, then 3,200 rpm for 3 min	Tricell kit		
Rossano 2017	16 ml	1,500 g 5 min	RegenKit-BCT-3, calcium gluconate per 0.9 ml of PRP 1:10 ratio	Sodium citrate	Mean platelet counts were1,9*105 in whole blood and 5.5*105 platelets/µl in PRP, 5 TIMES, 0.1ml/m2
Schiavone 2018	60–120 ml	Soft-spin, 1,500r 5 min	GLO PRP kit, and C-PunT	ACD-A	4.5 fold
Sclafani 2014	18 ml	1,100 g for 6 min	Calcium chloride	Thixotropic separator gel	
Shapiro 2020	10 ml	1,500 g 5 min	Thicotropic gel for separating		
Siah 2020	20 ml	300 g 18C, 5 min, 700 g for 17 min			
Starace 2019	10 ml	2,500 rpm for 10 min		ACD-A acid-citrate-dextrose	
Takikawa 2011	15 ml	1,700 r, 15 min, then 3,000 rpm in 5 min		0.2% sodium citrate	Platelet concentration in PRP (88.2 7 21.7 ? 104/1 mL, *n* = 15 persons) was significantly higher than that in whole blood (14.4 7 3.8 ? 104/1 mL, *n* = 15 persons)
Tawfik 2018	10 ml	1,200 g for 15 min, then 200 g for 10 min	1:9 ratio, 0.1 ml calcium gluconate per 0.9 ml of PRP	Sodium citrate	
Zolfaghari 2020	40 ml	Women: 1,400 r 14 m, men 1,600 r, then 4 min at 4,000 rpm	PRGF activbitor	3.8% sodium citrate	
Zhang 2018	16 ml	1,500 G, 3,000 r, 10 min			
Navarro 2015	18 ml	580 g for 8 min	PRGF activbitor	3.8% sodium citrate	
Zhang 2020	30 ml	3,000 r/min, 10 min	10% calciun cholirade	Sodium citrate	6.34 ± 0.4 fold

### Treatment Strategy

Despite all studies performed intradermal injections on the local alopecia area for AGA treatment, the whole procedure diverged (Table 3). 70% alcohol, spirit, or 0.1% octenidine hydrochloride spray were used for cleaning the local skin, and local anesthesia with the help of 2% lidocaine with 0.001% epinephrine were commonly used. two studies used anesthesia cream, one used soft head message, and one conducted cold air anesthesia before injections.

**TABLE 3:** Treatment protocols.

Author/year	PRP-injected (total, each injection) injection details	Treatment session arrangement		Anesthesia
Abaroa 2016	Total 3–5 ml to right half of the scales, intradermally, each puncture was made in spaces of 1cm, apply 0.2 ml in each puncture for 15 to 20 punctures	Twice a week for three weeks	At 72-h intervals over a three-week period	Not mentioned
Alves 2016	4 selected areas with 30-G needle, about 0.15 ml/cm^2^	1 month from each other for 3 months		None
Alves 2018	4 selected areas with 30-G needle, about 0.15 ml/cm^2^	1 month from each other for 3 months		None
Anitua 2017	3–4 cm^3^ of freshly activated RPGF injected to the hair-depleted area, with a 30-G needle	Every months for 3 sessions, 2 additional reminer injection doses were administered at months 4 and 7 after the start point		Not mentioned
Bruce 2019	The patient’s scalp was cleaned with 70% alcohol, and a grid was marked with dots approximately 1–2 cm apart, covering the affected area. A total of 5 ml of PRP was injected using a 30-gauge needle, approximately 0.1 ml per injection site and 50 injection points in the grid. The needle was angled close to 90°, targeting the transition between the dermis and subcutaneous layer			Cold air
Butt G 2020	3 ml PRP at 0.5 interval intradermally with insulin syringes	2 sessions each after 4 weeks		Local anesthetic gel, 1 h before giving injections
Butt G 2019	Cleaned with spirit, 1 cm distance using nappage technique for 1.5–2.5 mm deep in the skin	Repeated after 4 weeks		Local anesthetic gel, 1 h before giving injections
Dina 2019	4–5 ml of PRP to the scalp vertex and temporal hairline	3 treatments, spaced 4 weeks apart		Not mentioned
Dubin 2020	4.0 ml of PRP was injected 3–8 mm below the skin surface into the subdermal plane via a 30-gauge 0.5-inch needle. Each injection was comprised of 0.2 ml of PRP and spaced 1–2 cm apart	At week 0, 4, 8		Not mentioned
El-husseiny 2020	Multiple small ingections in a linear pattern 1 cm apart over the right half of the scalp, intradermally	3 treatments	3 weeks apart	Not mentioned
Gentile a 2020	0.2 ml*cm^2^ with a 30-G needle and10 ml luer lock syringe for 5 mm deep			Not mentioned
Gentile b 2020	0.2 ml*cm^2^ with 0.5 mm sterilemicro-needling procedure	Repeated every 15 days for three times		None
Gkini 2014	Cleaned using 0.1% octenidine hydrochloride spray, PRP for 0.05–0.1 ml/cm^2^ with a 27-G needle for 1.5–2.5 mm deep	3 treatment sessions with an interval of 3 weeks, a booster in 6 m		Local anethesia
Hausauer 2018	0.2–0.5 ml, half-inch needle every 2–3 cm at balding areas with a 32-gauge needle, half-inch needle deep subdermally	group1: Received 4 total injections, the first 3 at monthly intervals and the last 3 months later, group2: Received 2 total injections, one at baseline and one at 3 months		23% topical lidocaine or 7% tetracaine ointment
Ho 2020	0.1 ml PRP and spaced 1 cm apart for approximately 1 cm deep	Monthly for four additional months for four followed by maintenance injections every 3–6 months		Not mentioned
Juhasz 2020	0.1 ml PRP and spaced 1 cm apart for approximately 1 cm deep	2 PRP sessions completed at 4–6 weeks intervals		
Kang 2014	Cleaned with 70% alcohol and interfollicular injection	3 months interval		2% lidocaine with 0.001% epinephrine 3–5 ml injection
Laird 2018	Using microneedle on the treatment area with derma rollar for a 22-G gauge needle deep dermis/upper cutis			2% lidocaine with 0.001% epinephrine 3–5 ml injection
Lee 2015	Cleaned with 70% alcohol, intra-perifollicular injection	PDNR weekly for 12 weeks		2% lidocaine with 0.001% epinephrine 3–5 ml injection
Makki 2020	Cleaned and sterilized with spirit and povidone-iodine, 1 cm distance using nappage technique with the insulin syringe	4 times at 4 weeks interval		Topical anesthesia
Paththinige 2018	Loaded with 1 ml syringes before injection, cleaned with 70% alcohol, nappage technique (injections 1 cm apart, in a linear manner). 1.5–2.5 mm deep, with a 25-G needle	4 treatment sessions with initial three treatments in an interval of 3 weeks and a booster session performed at 14 weeks from the baseline treatment (2 months after the 3rd treatment session)		Local anethesia cream
Puig 2016				Anesthetized using a ring block method:achieved by injecting a 50:50 mixture of 2% lidocaine and 0.5% bupivicaine
Qu 2019	With a 30-G needle	With a 1-month interval for 6 consecutive sessions		Not mentioned
Rossano 2017	Clean with 0.1% citidine chloride spray, injected with a 27-G needle and 1 ml syringes for 1.5–2.5 mm deep in the skin	4sessions of PRP application each 40–60 days		Not mentioned
Schiavone 2018	1–2 mm deep	2 injection regimen with a 3-month interval between the 2 interventions		Not mentioned
Schiavone 2014	4 injections would be on the vertices of a square with sides = 1 cm. The amount injected, per each injection, was approximately 0.2–0.3 ml. With a 22–24G needle for 1 mm deep	2 injection regimen with a 3-month interval between the 2 interventions		Not mentioned
Sclafani 2014	0.1 ml per injection spot, separate by 5–8 mm intradermally	Every 4weeks for 3 treatment sessions		Not mentioned
Shapiro 2020	3–4 mm deep for 0.1–0.2 ml per injection/cm^2^	1-month intervals for 3 month with a final follow-up visit three months after the last treatment		Not mentioned
Siah 2020	3 cm^2^ area with a volume of 0.1 ml/cm^2^	5 injections with a 2-week interval		Not mentioned
Singhal 2015	8–12 ml of total volume	3 months at an interval of 2–3 weeks. The treatment is repeated every 2 weeks for four sessions		Not mentioned
Starace 2019	1 ml per injection point, with a 25G needle	Every 2 weeks for a total of 4 sessions		Anethesia cream
Takikawa 2011	A 25G needle	5 injections at 0,2,4,6,9 weeks		Not mentioned
Tan 2019		4 treatment sessions total 3 weeks apart for 9 weeks		Not mentioned
Zhang 2018	4 injections would be on the vertices of a square with sides = 1 cm. The amount injected, per each injection, was approximately 0.2–0.3 ml	Once a month, for 4 times		Not mentioned
Navarro 2015	A 30-G needle for total 2.5–3 ml, with a mesotherapy gun	2 treatment sessions with 1 month of time period between them		Soft head message
DeVasconcelos 2015	Intradermally at a dose of 0.2 ml of on each point of the affexted region, with spaces of approximately 2 cm between these points with a 26G 1/2 needle	3 injextions at 21 intervals		Not mentioned
Yang 2020	Inject with a 3 mm interval			
Zhang 2020	35 spot for 0.1 ml each spot	Once a month for 4times		Not mentioned

The majority of studies selected 22- to 30-G gauge needles with insulin syringes to perform the procedure, while sterilemicro-needling and DHN1 mesotherapy gun were also used in independent studies. Nappage technique (Modarressi, 2013) was taken by most studies with 1 to 3 cm distance between each injection point. 1cc PRP was injected within each grided injection point. The depth of intradermal injections was approximately 1.5–2.5 mm deep, but 0.5 mm deep for the sterilemicro-needling procedure. Intrafollicular injections and intra-perifollicular injections were also carried in several studies.

For treatment schedules, normally 3–5 treatment sessions with 4–6 intervals were performed. And the time of follow-up was commonly from 12 weeks to 9 months depending on the growth period of hairs (Lee et al., 2020).

### Outcome Evaluation Methods and Adverse Effects

In addition to Ludwig and Sinclair scale, endpoints evaluation methods included biopsy with ki-67 immunochemistry stain, photographic evaluation, hair density, hair count, global photographs, and phototrichogram analysis (Ueki et al., 2003), physician global assessment score (PhGAS), patient global assessment score (PaGAS), and pull test. The satisfaction questionnaires and scales were taken from the perspective of patients and other observers were also used to evaluate the efficacy of PRP in some of the recruited studies (Table 4).

**TABLE 4:** Outcomes measurement and adverse events in enrolled studies, and the evaluation of growth factors.

Author/year	Outcomes result and measurement	Adverse events	Outcomes on molecular levels
Abaroa 2016	Biopsy and photographs at week 12	Not mentioned	Not mentioned
Alves 2016	Global photographs and phototrichogram analysis (vertex, grontal, and occipital)	Not mentioned	Not mentioned
Alves 2018	Global photographs and phototrichogram analysis (vertex, grontal, and occipital)	Not mentioned	Not mentioned
Anitua 2017	Phototrichogram analysis using the TrichoScope ASG and the TrichoSciencePro hair and scalp diagnostic software, for women, mild scalp and crown region, hair density, hair diameterm terminal/vellus-like hair ratio and thin/regular/thick hair shafts among terminal follicles, and standardized global macrophotographs, self-assessment questionnaire and rated satisfaction following a likert scale, biosy from 6 volunteers, with ki-67	Not mentioned	Measuring TGF*β*1, PDGF-AB, EGF, VEGF, TSP-1 and Ang-1 increasing by ELISA
Bruce 2019	TrichoScan analysis and TrichoScan digital image analysis, hair count, hair density, cumulative thickness. QOL questionaire	weeks 4,8 and 12 of PRP treatment	Not mentioned
Butt G 2020	Macroscopic photographs, pull test, trichoscopic photomicrographs, physician glob asseement score (PhGAS), patient global assessment score (PaGAS), hair density	Not mentioned	Not mentioned
Butt G 2019	Macroscopic photographs, pull test, trichoscopic photomicrographs, physician glob asseement score (PhGAS), patient global assessment score (PaGAS)	Not mentioned	Not mentioned
Dubin 2020	Hair density, caliber and blinded global photographic assessment	In PRP, mild headache 50%, scalp tightness 50%, swelling29, redness 14%, post-injection bleeding 7%, and tingling 7%	Not mentioned
El-husseiny 2020	Patients' global photographs, Lugwig's and Sinclair's grading, questionaire of satisfacition, improvement in hair density, hair quality and pain injection and infection	11 headache, all reported mild pain during injections	No significant difference was found between the median concentrations (ng/L) of VEGF in both nonactivated and activated single- and double-spin prepared PRP measuring by ELISA
Garg 2017	Parameters which were observed on video-microscopy are hair count, diameter of hair, change in texture, multiplicity of hair, perifollicular halo, perifollicular pigmentation, increase in telogen hair and increase in vellus hair count	Not mentioned	Not mentioned
Gentile a 2020	Photography, physician's and patient's global assessment scale, and standardized phototrichograms	Not mentioned	Not mentioned
Gentile b 2020	Hair density by trichoscan, bioposi on anti-ki-67, anti-CD31	Not mentioned	Not mentioned
Gkini 2014	Hair density and patients' satisfaction, hair-pull test, dermoscopic photomicrographs, macroscopic photographs and a satisfaction questionnaire	25% mild pain after application, 60% scalo sensitivity	Not mentioned
Hausauer 2018	Folliscope and global photography, hair count, hair density, shaft caliber, Norwood-Hamilton or ludwig scale were determined, patient's satisfiaction	Not mentioned	Not mentioned
Ho 2020	Hair density and diameter using folliscope	Not mentioned	Not mentioned
Juhasz 2020	Hair density	Not mentioned	Not mentioned
Kang 2014	Phototrichogram scalp on hair numbers, thickness with follioscope	Not mentioned	Flow cytometry was performed using PRP preparation and an equal amount of peripheral blood in two healthy volunteers. One participant presented 6.7 cells ⁄ lL of CD34 ^+^ cells in peripheral blood, whereas those in the autologous PRP preparation were 31.1 cells ⁄ lL
Laird 2018	Satisfaction	Not mentioned	Not mentioned
Lee 2015	Hair density, hair count, hair thickness	Not mentioned	Western blot analyses of PDGF-A revealed significant differences between PRP-treated skin samples and control skin samples
Makki 2020	Photographs, quartile grading scale with two dermatologists, hair parameters, and hair density, patients satisfaction	Local injection pain	Not mentioned
Paththinige 2018	Hair density, hair count under dermoscopic photographs	Not mentioned	Not mentioned
Puig 2016	Hair count through photography, hair mass index (measured using the cohen hair check system); and patient survery	Not mentioned	Not mentioned
Qu 2019	Global macroscopic photographs, standardized phototrichograms, hair-pull test and satisfaction questionaire	Mild pain when injection, headache	Not mentioned
Rossano 2017	Gene type	Not mentioned	It showed a negative correlation. IL-1a could be used as a prognostic value for PRP efficacy in female pattern hair loss
Schiavone 2018	Photographs by global physician assessment (GPA) score and questionaires	Bruise after 48–72 h and spontaneously resolved in the fourth to fifth postop day	Not mentioned
Schiavone 2014	Photographs by global physician assessment (GPA) score and questionaires	Not mentioned	Not mentioned
Sclafani 2014	Hair density	No	Not mentioned
Shapiro 2020	Hair density	No	Not mentioned
Siah 2020	Dermatoscope, photography, hair density counting and hair caliber measurement	At week8, treatment site having a higher hair density 129.3 comparing to placebo site 115.3	PDGF-BB was the highest concentration of growth factor injected, and VEGF was tested for the lowest growth factor concentration
Singhal 2015	Hair count, hair thickness, hair root strength, and overall alopecia	3 mild head pain	Not mentioned
Starace 2019	Pull test, blobal photographs, and trichoscan, hair measurement	Not mentioned	Not mentioned
Takikawa 2011	Histological exam	Not mentioned	Not mentioned
Tan 2019	Folliscope, questionnaire	Not mentioned	Not mentioned
Tawfik 2018	Hair growth, hair density, hair diameter, photography, hair-pull test, patient's satisfaction scale, standardized phototrichograms, and patient’s satisfaction	Not mentioned	Not mentioned
Zhang 2018	Biopsy ki-67, hair density	Not mentioned	Not mentioned
Navarro 2015	Trichogram, photograpy, anagen, telogen	Not mentioned	Not mentioned
DeVasconcelos 2015	Mann-whitnety test	No	Not mentioned
Yang 2020	Dermascopy and photograph	Not mentioned	Not mentioned
Zhang 2020	Photographs, satisfaction questionaire	Not mentioned	Not mentioned
Zolfaghari 2020	Hair number and thickness	Not mentioned	Measuring TGF*β*1, TGF*β*2, PDGF, EGF, VEGF, and HGF increasing by ELISA

All-included studies showed positive responses and an improvement compared with the baseline; a few pooled patients reported adverse effects, including bruise and mild pain on injection sites after 48–72 h, which would resolve spontaneously in the 4th or 5th postoperation day. Mild headache and scalp sensitivity were also reported in a few studies.

### Labs

Apart from clinical evaluation, 7 out of 42 studies also did an assessment on platelet-rich growth factors (PRGF) (Table 4). 6 out of 7 focused on hair follicles regenerative factors including VEGF, PDGF, IGF, TGF-*β*, and HGF through enzyme-linked immunosorbent assay (ELISA), Western blot for mRNA expression, flow cytometry on CD34^+^, and animal models. One study reveals a negative correlation between individual genetic inflammatory profile and efficacy of PRP, which was different from PRP in males (Rossano et al., 2017).

### Subgroup Meta-Analysis

**FIGURE 5:**
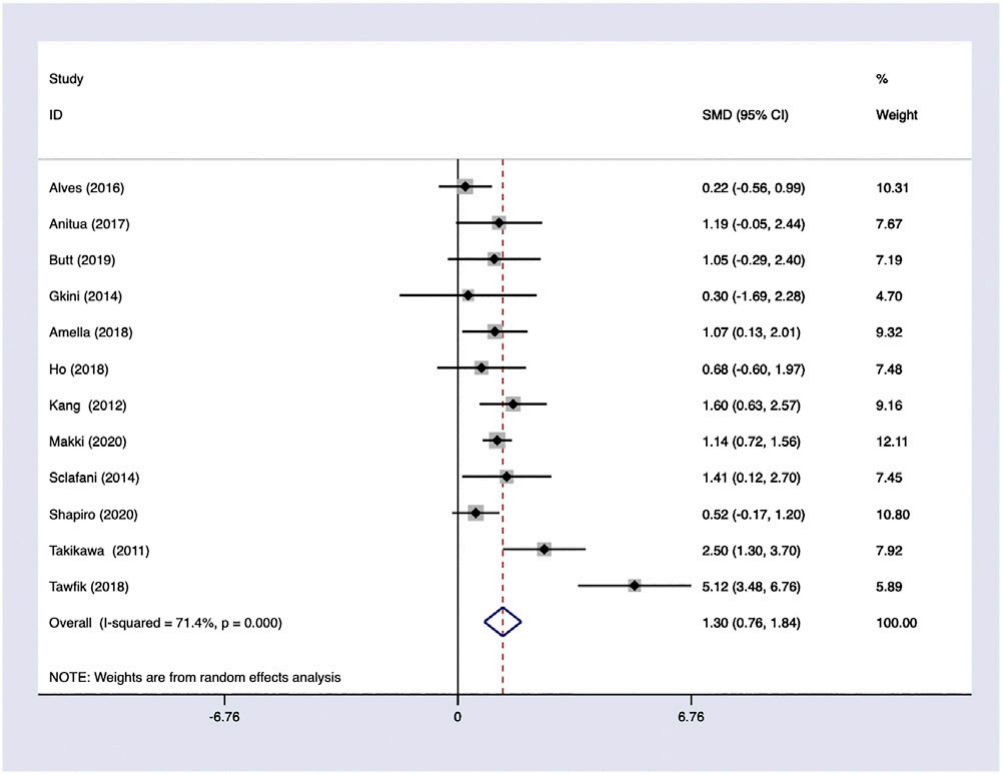
PRP efficacy compared to the baseline.

*PRP efficacy compared to placebo.* Only endpoints evaluated in the same measurement methods would be pooled in the meta-analysis. Using the random-effects model, PRP showed positive efficacy in the treatment of FPHL in hair density comparing to the control groups with odds ratio [OR] 1.61; 95% CI 0.52–2.70 (Figure 5).

**FIGURE 6:**
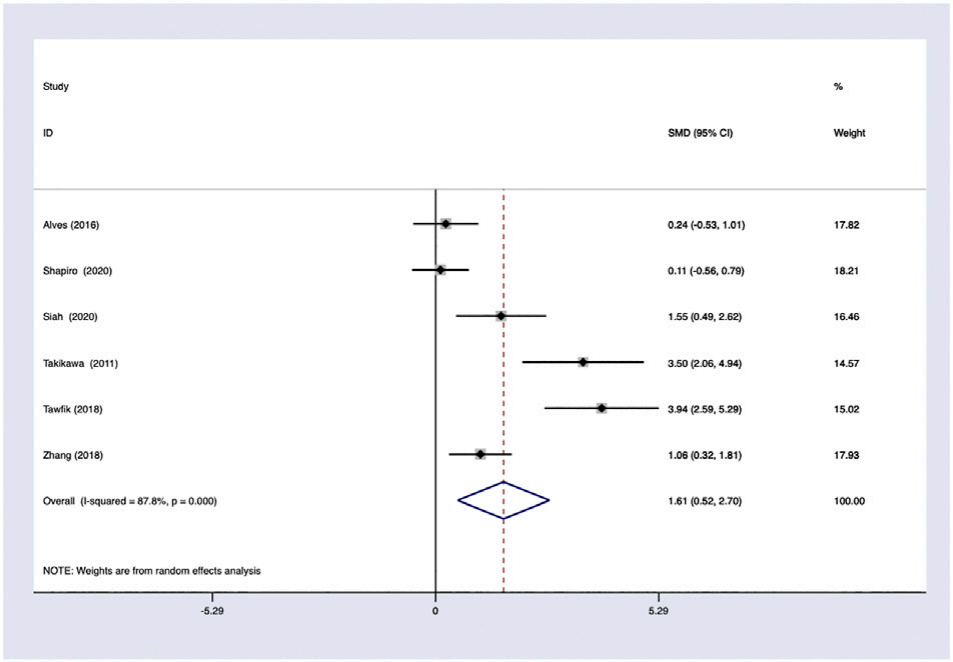
PRP efficacy compared to placebo.

*PRP efficacy compared to the baseline.* Only studies with high quality would be pooled in the analysis. Using a random-effects model, PRP showed effectiveness and improvements on hair density comparing to baseline with OR 1.11; 95% CI 0.86–1.37 (Figure 6).

Meta-analysis with other evaluation methods was not conducted due to limited studies.

### Heterogeneity

**FIGURE 7:**
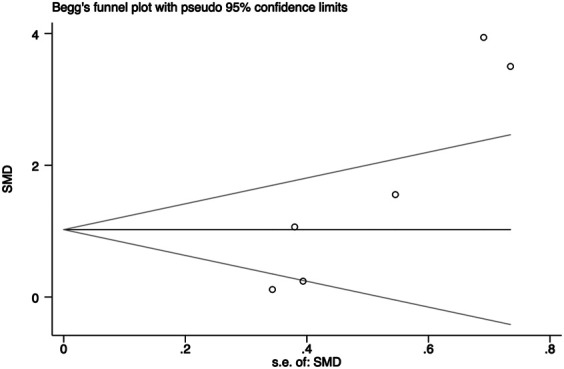
Begg’s funnel plot of studies compared the efficacy complared to placebo.

**FIGURE 8:**
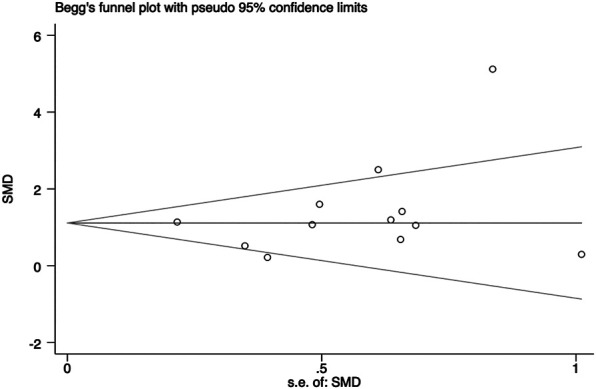
Begg’s funnel plot of studies compared the efficacy complared to the baseline.

**FIGURE 9:**
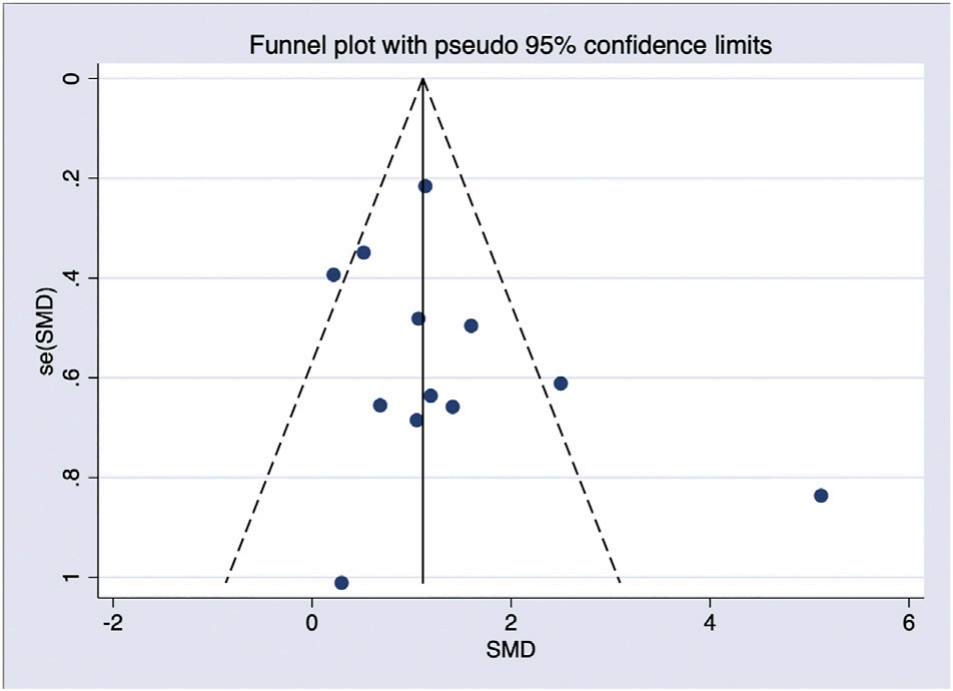
Funnel plot of studies compared the efficacy complared to the baseline.

In general, a meta-analysis of PRP efficacy comparing to the placebo had considerable statistical heterogeneity (I (Trueb, 2002) >75%), while a smaller substantial heterogeneity exists when comparing to the baseline. Visual inspection of funnel plots and Eggers tests were consistently in agreement with publication bias (Figure 7). Funnel plot that comparing with placebo suggested a tendency toward lack of large studies with both positive and negative results, two of the enrolled small studies showed little association (Figure 8). For the funnel plot in the baseline of the comparison group, a tendency toward a lack of small studies with negative and positive results was indicated (Figure 9).

## Discussion

Our study demonstrated an effective response and relative safety for the application of PRP in the treatment of FPHL with the meta-analysis for RCT and observation studies. Especially for those patients with negative response to the tropical use of minoxidil, PRP offered an alternative treatment option. We only used hair density as an endpoint to perform meta-analysis for the diversity of endpoint settings among the enrolled studies, while hair density is the most commonly used one. This result is in accordance with previous systemic review (Torabi et al., 2020).

The major strengths of the study suggested a positive efficacy of PRP for female AGA and elucidated different PRP preparing methods and treatment regimes. Although there were several systemic reviews (Torabi et al., 2020) and meta-analysis on the application of PRP in AGA treatment (Evans et al., 2020), one mainly focus on female patients remain scarce.

In the author’s opinion, the need for larger scale randomized–controlled trials and extensive meta-analysis precedes a considerable heterogeneity challenge. Heterogeneity anticipated was mainly because of female’s nationalities and races, the difference treatment regime, PRP preparation methods, injection details, and PRP concentration. Subgroup analysis according to the different patient’s races and nationalities were not included in this study for the limited-published data and only a few studies included races information. Lower heterogeneity showed when studies of low quality were abandoned suggesting methodological heterogeneity contributed. Although efforts had been made to accommodate this issue, limited number of studies just based on women patients impeded methods like subgroup analysis, which resulted in a challenge to interpret the efficacy of PRP on female patients. As some studies measured patients of both gender but without separated data for female patients, we cannot prove strong evidence for the treating efficacy.

Selection bias is possibly addressed by group settings among studies. The prevalence of AGA is known to vary from the races (Paik et al., 2001; Gan and Sinclair, 2005; Khumalo et al., 2007). Enrolled studies were conducted by different countries, while all-pooled female participants were diverse in races. Subgroups regarding to ethnicity were not included in this study for the limited-enrolled studies. Whether divergences exist in the efficacy of PRP through races remain unclear. Besides, the majority of randomized studies were set in half-headed. Patients received PRP on half-scalp and placebo on the other half. Both injected spots showed improvement of hair growth or hair density. Although PRP-injected site showed a more obvious effect, the improvement of hair density on the placebo-injected sites may result in a smaller difference between PRP and placebo. Besides, whether PRP had a growth effect on the opposite side of the scalp remains obscure.

In aggregate, our study showed an efficacy of PRP in the therapy of FPHL through hair density evaluation. Although the mechanism of action on hair follicles is still under debate, it has proved to be a promising option for FPHL treatment. Given that current treatments differ from methodology and treatment technique, further studies are needed to define standardized protocols and large-scale–randomized trials still need to be conducted to confirm its efficacy.

## Data Availability

The original contributions presented in the study are included in the article/Supplementary Material; further inquiries can be directed to the corresponding author.
