# Supplementary material for: Platelet-Rich Plasma in Female Androgenic Alopecia: A Comprehensive Systematic Review and Meta-Analysis
Source: Front Pharmacol. 2021 May 6;12:642980. doi: 10.3389/fphar.2021.642980 (PMC8204330; doi:10.3389/fphar.2021.642980)
Supplement: Supplementary file 1 [file DataSheet1.docx]

Supplementary Material

# Supplementary Data

1. Abaroa F, Reyes K, Barrera D, Castelán E, Montemayor B, Izabal G, et al. Histological findings of follicular units in patients with androgenetic alopecia before and after application of autologous platelet-rich plasma. Dermatologia Revista Mexicana. 2016

2. Alves R, Grimalt R. Platelet-Rich Plasma in Combination With 5% Minoxidil Topical Solution and 1 mg Oral Finasteride for the Treatment of Androgenetic Alopecia: A Randomized Placebo-Controlled, Double-Blind, Half-Head Study. Dermatologic Surgery. 2018

3. Anitua E, Pino A, Martinez N, Orive G, Berridi D. The Effect of Plasma Rich in Growth Factors on Pattern Hair Loss: A Pilot Study. Dermatologic Surgery. 2017

4. Bruce A, Pincelli T, Heckman MG, Desmond CM, Arthurs JR, Diehl NN, et al. A randomized, controlled pilot trial comparing platelet- rich plasma to topical minoxidil foam for treatment of androgenic alopecia in women. Journal of the American Academy of Dermatology. 2019

5. Butt G, Hussain I, Ahmad FJ, Choudhery MS. Stromal vascular fraction-enriched platelet-rich plasma therapy reverses the effects of androgenetic alopecia. Journal of Cosmetic Dermatology. 2020

6. Butt G, Hussain I, Ahmed FJ, Choudhery MS. Efficacy of platelet-rich plasma in androgenetic alopecia patients. Journal of Cosmetic Dermatology. 2019

7. Dina Y, Aguh C. Use of Platelet-Rich Plasma in Cicatricial Alopecia. Dermatol Surg. 2019

8. Dubin DP, Lin MJ, Leight HM, Farberg AS, Torbeck RL, Burton WB, et al. The effect of platelet-rich plasma on female androgenetic alopecia: A randomized controlled trial. Journal of the American Academy of Dermatology. 2020

9. El-Husseiny RM, Saleh HM, Moustafa AA, Salem SA. Comparison between single- versus double-spin prepared platelet-rich plasma injection in treatment of female pattern hair loss: clinical effect and relation to vascular endothelial growth factor. Arch Dermatol Res. 2020

10. Farris PK, Rogers N, McMichael A, Kogan S. A Novel Multi-Targeting Approach to Treating Hair Loss, Using Standardized Nutraceuticals. Journal of Drugs in Dermatology. 2017

11. Ferrando J, García-García SC, González-de-Cossío AC, Bou L, Navarra E. A Proposal of an Effective Platelet-rich Plasma Protocol for the Treatment of Androgenetic Alopecia. Int J Trichology. 2017

12. Garg S, Manchanda S. Platelet-rich plasma-an 'Elixir' for treatment of alopecia: Personal experience on 117 patients with review of literature. Stem Cell Investigation. 2017

13. Gentile P, Dionisi L, Pizzicannella J, de Angelis B, de Fazio D, Garcovich S. A randomized blinded retrospective study: the combined use of micro-needling technique, low-level laser therapy and autologous non-activated platelet-rich plasma improves hair re-growth in patients with androgenic alopecia. Expert Opinion on Biological Therapy. 2020

14. Gentile P, Garcovich S. Autologous activated platelet-rich plasma (AA-PRP) and non-activated (A-PRP) in hair growth: a retrospective, blinded, randomized evaluation in androgenetic alopecia. Expert Opinion on Biological Therapy. 2020

15. Gentile P, Garcovich S, Scioli MG, Bielli A, Orlandi A, Cervelli V. Mechanical and Controlled PRP Injections in Patients Affected by Androgenetic Alopecia. Jove-Journal of Visualized Experiments. 2018

16. Gkini MA, Kouskoukis AE, Tripsianis G, Rigopoulos D, Kouskoukis K. Study of platelet-rich plasma injections in the treatment of androgenetic alopecia through an one-year period. J Cutan Aesthet Surg. 2014

17. Hausauer AK, Jones DH. Evaluating the Efficacy of Different Platelet-Rich Plasma Regimens for Management of Androgenetic Alopecia: A Single-Center, Blinded, Randomized Clinical Trial. Dermatologic Surgery. 2018

18. Ho A, Sukhdeo K, Lo Sicco K, Shapiro J. Trichologic response of platelet-rich plasma in androgenetic alopecia is maintained during combination therapy. Journal of the American Academy of Dermatology. 2020

19. Juhasz MLW, Sukhdeo K, Lo Sicco K, Shapiro J. Stratifying clinical response to adjuvant platelet-rich plasma in patients with androgenetic alopecia. British Journal of Dermatology. 2020

20. Kang JS, Zheng Z, Choi MJ, Lee SH, Kim DY, Cho SB. The effect of CD34+cell-containing autologous platelet-rich plasma injection on pattern hair loss: a preliminary study. Journal of the European Academy of Dermatology and Venereology. 2014

21. Laird ME, Lo Sicco KI, Reed ML, Brinster NK. Platelet-Rich Plasma for the Treatment of Female Pattern Hair Loss: A Patient Survey. Dermatologic Surgery. 2018

22. Lee SH, Zheng ZL, Kang JS, Kim DY, Oh SH, Cho SB. Therapeutic efficacy of autologous platelet-rich plasma and polydeoxyribonucleotide on female pattern hair loss. Wound Repair and Regeneration. 2015

23. Makki M, Younes AKH, Salah I. Evaluation of the effect of autologous platelet-rich plasma on androgenic alopecia. Journal of Cosmetic and Laser Therapy. 2020

24. Paththinige N, Akarawita J, Jeganathan G. The clinical efficacy and safety of autologous activated platelet rich plasma injection (AA-PRP) in androgenic alopecia (AGA). Australasian Journal of Dermatology. 2018

25. Puig CJ, Reese R, Peters M. Double-Blind, Placebo-Controlled Pilot Study on the Use of Platelet-Rich Plasma in Women With Female Androgenetic Alopecia. Dermatologic Surgery. 2016

26. Qu Q, Shi PL, Yi YH, Fan ZX, Liu XM, Zhu DC, et al. Efficacy of Platelet-rich Plasma for Treating Androgenic Alopecia of Varying Grades. Clinical Drug Investigation. 2019

27. Rossano F, Di Martino S, Iodice L, Di Paolo M, Misso S, Tomeo R, et al. Correlation between individual inflammation genetic profile and platelet rich plasma efficacy in hair follicle regeneration: a pilot study reveals prognostic value of IL-1 alpha polymorphism. European Review for Medical and Pharmacological Sciences. 2017

28. Schiavone G, Paradisi A, Ricci F, Abeni D. Injectable Platelet-, Leukocyte-, and Fibrin-Rich Plasma (iL-PRF) in the Management of Androgenetic Alopecia. Dermatologic Surgery. 2018

29. Schiavone G, Raskovic D, Greco J, Abeni D. Platelet-Rich Plasma for Androgenetic Alopecia: A Pilot Study. Dermatologic Surgery. 2014

30. Sclafani AP. Platelet-Rich Fibrin Matrix (PRFM) for Androgenetic Alopecia. Facial Plastic Surgery. 2014

31. Shapiro J, Ho A, Sukhdeo K, Yin L, Lo Sicco K. Evaluation of platelet-rich plasma as a treatment for androgenetic alopecia: A randomized controlled trial. Journal of the American Academy of Dermatology. 2020

32. Siah TW, Guo HW, Chu TM, Santos L, Nakamura H, Leung G, et al. Growth factor concentrations in platelet-rich plasma for androgenetic alopecia: An intra-subject, randomized, blinded, placebo-controlled, pilot study. Experimental Dermatology. 2020

33. Singhal P, Agarwal S, Dhot PS, Sayal SK. Efficacy of platelet-rich plasma in treatment of androgenic alopecia. Asian J Transfus Sci. 2015

34. Starace M, Alessandrini A, D'Acunto C, Melandri D, Bruni F, Patrizi A, et al. Platelet-rich plasma on female androgenetic alopecia: Tested on 10 patients. Journal of Cosmetic Dermatology. 2019

35. Takikawa M, Nakamura S, Nakamura S, Ishirara M, Kishimoto S, Sasaki K, et al. Enhanced effect of platelet-rich plasma containing a new carrier on hair growth. Dermatol Surg. 2011

36. Tan CH, Lee JSS, Tan KT, Wang ECE, Chan RKW, Chuah SY. A randomized double-blind, split-scalp, placebocontrolled study to evaluate the efficacy of platelet-rich plasma for the treatment of androgenetic alopecia. British Journal of Dermatology. 2019

37. Tawfik AA, Osman MAR. The effect of autologous activated platelet-rich plasma injection on female pattern hair loss: A randomized placebo-controlled study. Journal of Cosmetic Dermatology. 2018

38. Zolfaghari M, Barzegar M, Amiri V, Allahbakhshian Farsani M, Jesri S, Kiani Harchegani A, et al. Standardization of Plasma Rich in Growth Factors (PRGF) and its effects on androgenic hair loss. J Cosmet Laser Ther. 2020

# Supplementary 1

Pub med: search history

1 Mesh:

Baldness OR Hair Loss OR Hair Losses OR Loss, Hair OR Losses, Hair OR Alopecia, Male Pattern OR Male Pattern Alopecia OR Baldness, Male Pattern OR Male Pattern Baldness OR Female Pattern Baldness OR Baldness, Female Pattern OR Androgenetic Alopecia OR Pattern Baldness OR Baldness, Pattern OR Androgenic Alopecia OR Alopecia, Androgenic OR Alopecias, Androgenic OR Androgenic Alopecias OR Alopecia, Androgenetic OR Pseudopelade OR Alopecia Cicatrisata OR Alopecia Cicatrisatas

Result:34,505

("alopecia"[MeSH Terms] OR "alopecia"[All Fields] OR "baldness"[All Fields] OR "balding"[All Fields]) OR ("alopecia"[MeSH Terms] OR "alopecia"[All Fields] OR ("hair"[All Fields] AND "loss"[All Fields]) OR "hair loss"[All Fields]) OR ("alopecia"[MeSH Terms] OR "alopecia"[All Fields] OR ("hair"[All Fields] AND "losses"[All Fields]) OR "hair losses"[All Fields]) OR ("alopecia"[MeSH Terms] OR "alopecia"[All Fields] OR ("loss"[All Fields] AND "hair"[All Fields]) OR "loss, hair"[All Fields]) OR ("alopecia"[MeSH Terms] OR "alopecia"[All Fields] OR ("losses"[All Fields] AND "hair"[All Fields]) OR "losses, hair"[All Fields]) OR ("alopecia"[MeSH Terms] OR "alopecia"[All Fields] OR ("alopecia"[All Fields] AND "male"[All Fields] AND "pattern"[All Fields]) OR "alopecia, male pattern"[All Fields]) OR ("alopecia"[MeSH Terms] OR "alopecia"[All Fields] OR ("male"[All Fields] AND "pattern"[All Fields] AND "alopecia"[All Fields]) OR "male pattern alopecia"[All Fields]) OR ("alopecia"[MeSH Terms] OR "alopecia"[All Fields] OR ("baldness"[All Fields] AND "male"[All Fields] AND "pattern"[All Fields]) OR "baldness male pattern"[All Fields]) OR ("alopecia"[MeSH Terms] OR "alopecia"[All Fields] OR ("male"[All Fields] AND "pattern"[All Fields] AND "baldness"[All Fields]) OR "male pattern baldness"[All Fields]) OR ("alopecia"[MeSH Terms] OR "alopecia"[All Fields] OR ("female"[All Fields] AND "pattern"[All Fields] AND "baldness"[All Fields]) OR "female pattern baldness"[All Fields]) OR ("alopecia"[MeSH Terms] OR "alopecia"[All Fields] OR ("baldness"[All Fields] AND "female"[All Fields] AND "pattern"[All Fields]) OR "baldness female pattern"[All Fields]) OR ("alopecia"[MeSH Terms] OR "alopecia"[All Fields] OR ("androgenetic"[All Fields] AND "alopecia"[All Fields]) OR "androgenetic alopecia"[All Fields]) OR ("alopecia"[MeSH Terms] OR "alopecia"[All Fields] OR ("pattern"[All Fields] AND "baldness"[All Fields]) OR "pattern baldness"[All Fields]) OR ("alopecia"[MeSH Terms] OR "alopecia"[All Fields] OR ("baldness"[All Fields] AND "pattern"[All Fields]) OR "baldness, pattern"[All Fields]) OR ("alopecia"[MeSH Terms] OR "alopecia"[All Fields] OR ("androgenic"[All Fields] AND "alopecia"[All Fields]) OR "androgenic alopecia"[All Fields]) OR ("alopecia"[MeSH Terms] OR "alopecia"[All Fields] OR ("alopecia"[All Fields] AND "androgenic"[All Fields]) OR "alopecia androgenic"[All Fields]) OR ("alopecia"[MeSH Terms] OR "alopecia"[All Fields] OR ("alopecias"[All Fields] AND "androgenic"[All Fields]) OR "alopecias, androgenic"[All Fields]) OR ("alopecia"[MeSH Terms] OR "alopecia"[All Fields] OR ("androgenic"[All Fields] AND "alopecias"[All Fields]) OR "androgenic alopecias"[All Fields]) OR ("alopecia"[MeSH Terms] OR "alopecia"[All Fields] OR ("alopecia"[All Fields] AND "androgenetic"[All Fields]) OR "alopecia androgenetic"[All Fields]) OR ("alopecia"[MeSH Terms] OR "alopecia"[All Fields] OR "pseudopelade"[All Fields]) OR ("alopecia"[MeSH Terms] OR "alopecia"[All Fields] OR ("alopecia"[All Fields] AND "cicatrisata"[All Fields]) OR "alopecia cicatrisata"[All Fields]) OR ("alopecia"[MeSH Terms] OR "alopecia"[All Fields] OR ("alopecia"[All Fields] AND "cicatrisatas"[All Fields]) OR "alopecia cicatrisatas"[All Fields])

2

Mesh:

Platelet-Rich Plasma

Result: 12,374

"platelet rich plasma"[MeSH Terms] OR ("platelet rich"[All Fields] AND "plasma"[All Fields]) OR "platelet rich plasma"[All Fields] OR ("platelet"[All Fields] AND "rich"[All Fields] AND "plasma"[All Fields]) OR "platelet rich plasma"[All Fields]

(#1) AND (#2)

Result: 228

Embase:

| No. | Query | Results |
| --- | --- | --- |
| #10 | #3 AND #9 | 145 |
| #9 | #4 OR #5 OR #6 | 13863 |
| #6 | 'platelet rich plasma gel'/exp | 10 |
| #5 | 'platelet-rich plasma cell'/exp | 307 |
| #4 | 'thrombocyte rich plasma'/exp | 13660 |
| #3 | 'male type alopecia'/exp | 3072 |

## Supplementary 2

| No. | Authors | Title | Journal name | year |
| --- | --- | --- | --- | --- |
| 1 | Abaroa F, Reyes K, Barrera D, Castelán E, Montemayor B, Izabal G, et al. | Histological findings of follicular units in patients with androgenetic alopecia before and after application of autologous platelet-rich plasma. | Dermatologia Revista Mexicana. | 2016 |
| 2 | Alves R, Grimalt R. | Randomized Placebo-Controlled, Double-Blind, Half-Head Study to Assess the Efficacy of Platelet-Rich Plasma on the Treatment of Androgenetic Alopecia. | Dermatol Surg. | 2016 |
| 3 | Alves R, Grimalt R. | Platelet-Rich Plasma in Combination With 5% Minoxidil Topical Solution and 1 mg Oral Finasteride for the Treatment of Androgenetic Alopecia: A Randomized Placebo-Controlled, Double-Blind, Half-Head Study. | Dermatologic Surgery. | 2018 |
| 4 | Anitua E, Pino A, Martinez N, Orive G, Berridi D. | The Effect of Plasma Rich in Growth Factors on Pattern Hair Loss: A Pilot Study. | Dermatologic Surgery. | 2017 |
| 5 | Bruce A, Pincelli T, Heckman MG, Desmond CM, Arthurs JR, Diehl NN, et al. | A randomized, controlled pilot trial comparing platelet- rich plasma to topical minoxidil foam for treatment of androgenic alopecia in women. | Journal of the American Academy of Dermatology. | 2019 |
| 6 | Butt G, Hussain I, Ahmad FJ, Choudhery MS. | Stromal vascular fraction-enriched platelet-rich plasma therapy reverses the effects of androgenetic alopecia. | Journal of Cosmetic Dermatology. | 2020 |
| 7 | Butt G, Hussain I, Ahmed FJ, Choudhery MS. | Efficacy of platelet-rich plasma in androgenetic alopecia patients. | Journal of Cosmetic Dermatology. | 2019 |
| 8 | Dina Y, Aguh C. | Use of Platelet-Rich Plasma in Cicatricial Alopecia. | Dermatol Surg. | 2019 |
| 9 | Dubin DP, Lin MJ, Leight HM, Farberg AS, Torbeck RL, Burton WB, et al. | The effect of platelet-rich plasma on female androgenetic alopecia: A randomized controlled trial. | Journal of the American Academy of Dermatology. | 2020 |
| 10 | El-Husseiny RM, Saleh HM, Moustafa AA, Salem SA. | Comparison between single- versus double-spin prepared platelet-rich plasma injection in treatment of female pattern hair loss: clinical effect and relation to vascular endothelial growth factor. | Arch Dermatol Res. | 2020 |
| 11 | Garg S, Manchanda S. | Platelet-rich plasma-an 'Elixir' for treatment of alopecia: Personal experience on 117 patients with review of literature. | Stem Cell Investigation. | 2017 |
| 12 | Gentile P, Dionisi L, Pizzicannella J, de Angelis B, de Fazio D, Garcovich S. | A randomized blinded retrospective study: the combined use of micro-needling technique, low-level laser therapy and autologous non-activated platelet-rich plasma improves hair re-growth in patients with androgenic alopecia. | Expert Opinion on Biological Therapy. | 2020 |
| 13 | Gentile P, Garcovich S. | Autologous activated platelet-rich plasma (AA-PRP) and non-activated (A-PRP) in hair growth: a retrospective, blinded, randomized evaluation in androgenetic alopecia. | Expert Opinion on Biological Therapy. | 2020 |
| 14 | Gentile P, Garcovich S, Scioli MG, Bielli A, Orlandi A, Cervelli V. | Mechanical and Controlled PRP Injections in Patients Affected by Androgenetic Alopecia. | Jove-Journal of Visualized Experiments. | 2018 |
| 15 | Gkini MA, Kouskoukis AE, Tripsianis G, Rigopoulos D, Kouskoukis K. | Study of platelet-rich plasma injections in the treatment of androgenetic alopecia through an one-year period. | J Cutan Aesthet Surg. | 2014 |
| 16 | Hausauer AK, Jones DH. | Evaluating the Efficacy of Different Platelet-Rich Plasma Regimens for Management of Androgenetic Alopecia: A Single-Center, Blinded, Randomized Clinical Trial. | Dermatologic Surgery. | 2018 |
| 17 | Ho A, Sukhdeo K, Lo Sicco K, Shapiro J. | Trichologic response of platelet-rich plasma in androgenetic alopecia is maintained during combination therapy. | Journal of the American Academy of Dermatology. | 2020 |
| 18 | Juhasz MLW, Sukhdeo K, Lo Sicco K, Shapiro J. | Stratifying clinical response to adjuvant platelet-rich plasma in patients with androgenetic alopecia. | British Journal of Dermatology. | 2020 |
| 19 | Kang JS, Zheng Z, Choi MJ, Lee SH, Kim DY, Cho SB. | The effect of CD34+cell-containing autologous platelet-rich plasma injection on pattern hair loss: a preliminary study. | Journal of the European Academy of Dermatology and Venereology. | 2014 |
| 20 | Laird ME, Lo Sicco KI, Reed ML, Brinster NK. | Platelet-Rich Plasma for the Treatment of Female Pattern Hair Loss: A Patient Survey. | Dermatologic Surgery. | 2018 |
| 21 | Lee SH, Zheng ZL, Kang JS, Kim DY, Oh SH, Cho SB. | Therapeutic efficacy of autologous platelet-rich plasma and polydeoxyribonucleotide on female pattern hair loss. | Wound Repair and Regeneration. | 2015 |
| 22 | Makki M, Younes AKH, Salah I. | Evaluation of the effect of autologous platelet-rich plasma on androgenic alopecia. | Journal of Cosmetic and Laser Therapy. | 2020 |
| 23 | Navarro RM, Pino A, Martinez-Andres A, Molina C, Martinez AM, Martinez N, et al. | The effect of plasma rich in growth factors combined with follicular unit extraction surgery for the treatment of hair loss: A pilot study. | J Cosmet Dermatol. | 2018 |
| 24 | Paththinige N, Akarawita J, Jeganathan G. | The clinical efficacy and safety of autologous activated platelet rich plasma injection (AA-PRP) in androgenic alopecia (AGA). | Australasian Journal of Dermatology. | 2018 |
| 25 | Puig CJ, Reese R, Peters M. | Double-Blind, Placebo-Controlled Pilot Study on the Use of Platelet-Rich Plasma in Women With Female Androgenetic Alopecia. | Dermatologic Surgery. | 2016 |
| 26 | Qu Q, Shi PL, Yi YH, Fan ZX, Liu XM, Zhu DC, et al. | Efficacy of Platelet-rich Plasma for Treating Androgenic Alopecia of Varying Grades. | Clinical Drug Investigation. | 2019 |
| 27 | Rossano F, Di Martino S, Iodice L, Di Paolo M, Misso S, Tomeo R, et al. | Correlation between individual inflammation genetic profile and platelet rich plasma efficacy in hair follicle regeneration: a pilot study reveals prognostic value of IL-1 alpha polymorphism. | European Review for Medical and Pharmacological Sciences. | 2017 |
| 28 | Schiavone G, Paradisi A, Ricci F, Abeni D. | Injectable Platelet-, Leukocyte-, and Fibrin-Rich Plasma (iL-PRF) in the Management of Androgenetic Alopecia. | Dermatologic Surgery. | 2018 |
| 29 | Schiavone G, Raskovic D, Greco J, Abeni D. | Platelet-Rich Plasma for Androgenetic Alopecia: A Pilot Study. | Dermatologic Surgery. | 2014 |
| 30 | Sclafani AP. | Platelet-Rich Fibrin Matrix (PRFM) for Androgenetic Alopecia. | Facial Plastic Surgery. | 2014 |
| 31 | Shapiro J, Ho A, Sukhdeo K, Yin L, Lo Sicco K. | Evaluation of platelet-rich plasma as a treatment for androgenetic alopecia: A randomized controlled trial. | Journal of the American Academy of Dermatology. | 2020 |
| 32 | Siah TW, Guo HW, Chu TM, Santos L, Nakamura H, Leung G, et al. | Growth factor concentrations in platelet-rich plasma for androgenetic alopecia: An intra-subject, randomized, blinded, placebo-controlled, pilot study. | Experimental Dermatology. | 2020 |
| 33 | Singhal P, Agarwal S, Dhot PS, Sayal SK. | Efficacy of platelet-rich plasma in treatment of androgenic alopecia. | Asian J Transfus Sci. | 2015 |
| 34 | Starace M, Alessandrini A, D'Acunto C, Melandri D, Bruni F, Patrizi A, et al. | Platelet-rich plasma on female androgenetic alopecia: Tested on 10 patients. | Journal of Cosmetic Dermatology. | 2019 |
| 35 | Takikawa M, Nakamura S, Nakamura S, Ishirara M, Kishimoto S, Sasaki K, et al. | Enhanced effect of platelet-rich plasma containing a new carrier on hair growth. | Dermatol Surg. | 2011 |
| 36 | Tan CH, Lee JSS, Tan KT, Wang ECE, Chan RKW, Chuah SY. | A randomized double-blind, split-scalp, placebocontrolled study to evaluate the efficacy of platelet-rich plasma for the treatment of androgenetic alopecia. | British Journal of Dermatology. | 2019 |
| 37 | Tawfik AA, Osman MAR. | The effect of autologous activated platelet-rich plasma injection on female pattern hair loss: A randomized placebo-controlled study. | Journal of Cosmetic Dermatology. | 2018 |
| 38 | Zhang X, Gao H, Wang Z. | ClinicaI efficacy and mechanism of platelet-rich plasma in Patients with Androgenetic Alopecia. | J Med Ther & Prac. | 2018 |
| 39 | Zolfaghari M, Barzegar M, Amiri V, Allahbakhshian Farsani M, Jesri S, Kiani Harchegani A, et al. | Standardization of Plasma Rich in Growth Factors (PRGF) and its effects on androgenic hair loss. | J Cosmet Laser Ther. | 2020 |
| 40 | Navarro MR, Asín M, Martínez AM, Molina C, Navarro V, Pino A, et al. | Plasma rich in growth factors (PRGF) for the treatment of androgenetic alopecia. | European Journal of Plastic Surgery. | 2015 |
| 41 | Rossana Cantanhede Farias dV, Karla A, Géssica Cantadori Funes A, João Guilherme Finizola dV, Natalie Schnaider B. | The application of platelet-rich plasma in the treatment of androgenic alopecia. | Surgery Cosmetic Dermatololy. | 2015 |
| 42 | YANG Gy, REN Y-h, YANG Y-j. | Effect of platelet-rich plasma combined with topical Minodil tincture in the treatment of androgenetic alopecia. | Journal of Clinical and Experimental Medicine. | 2020 |

**
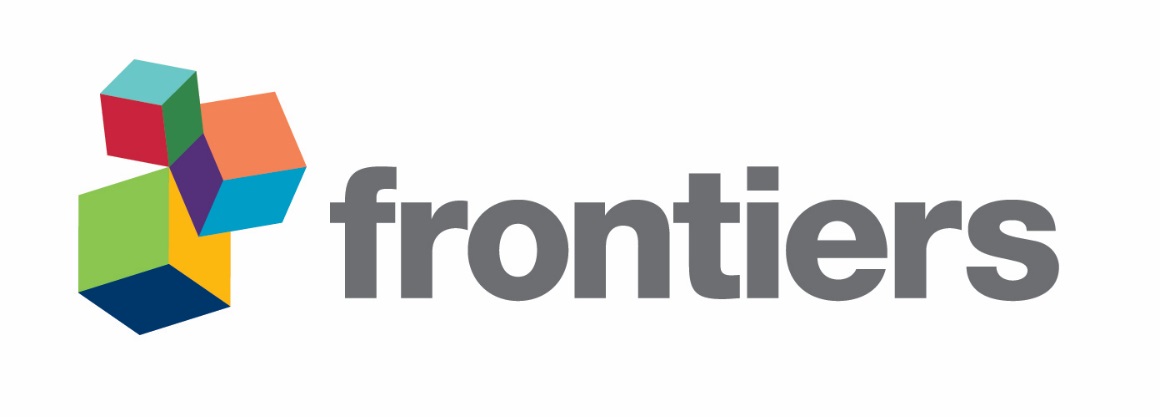
**

**Supplementary 3**

| **Section/topic** | **#** | **Checklist item** | **Reported on page #** |
| --- | --- | --- | --- |
| **TITLE** | | |  |
| Title | 1 | Identify the report as a systematic review, meta-analysis, or both. | 1 |
| **ABSTRACT** | | |  |
| Structured summary | 2 | Provide a structured summary including, as applicable: background; objectives; data sources; study eligibility criteria, participants, and interventions; study appraisal and synthesis methods; results; limitations; conclusions and implications of key findings; systematic review registration number. | 2 |
| **INTRODUCTION** | | |  |
| Rationale | 3 | Describe the rationale for the review in the context of what is already known. | 3 |
| Objectives | 4 | Provide an explicit statement of questions being addressed with reference to participants, interventions, comparisons, outcomes, and study design (PICOS). | 3 |
| **METHODS** | | |  |
| Protocol and registration | 5 | Indicate if a review protocol exists, if and where it can be accessed (e.g., Web address), and, if available, provide registration information including registration number. | 4 |
| Eligibility criteria | 6 | Specify study characteristics (e.g., PICOS, length of follow-up) and report characteristics (e.g., years considered, language, publication status) used as criteria for eligibility, giving rationale. | 4 |
| Information sources | 7 | Describe all information sources (e.g., databases with dates of coverage, contact with study authors to identify additional studies) in the search and date last searched. | 4 |
| Search | 8 | Present full electronic search strategy for at least one database, including any limits used, such that it could be repeated. | 4 |
| Study selection | 9 | State the process for selecting studies (i.e., screening, eligibility, included in systematic review, and, if applicable, included in the meta-analysis). | 4 |
| Data collection process | 10 | Describe method of data extraction from reports (e.g., piloted forms, independently, in duplicate) and any processes for obtaining and confirming data from investigators. | 4 |
| Data items | 11 | List and define all variables for which data were sought (e.g., PICOS, funding sources) and any assumptions and simplifications made. | 4-5 |
| Risk of bias in individual studies | 12 | Describe methods used for assessing risk of bias of individual studies (including specification of whether this was done at the study or outcome level), and how this information is to be used in any data synthesis. | 5 |
| Summary measures | 13 | 5 | 5 |
| Synthesis of results | 14 | Describe the methods of handling data and combining results of studies, if done, including measures of consistency (e.g., I^2^) for each meta-analysis. | 5 |

Page 1 of 2

| **Section/topic** | **#** | **Checklist item** | **Reported on page #** |  |
| --- | --- | --- | --- | --- |
| Risk of bias across studies | 15 | Specify any assessment of risk of bias that may affect the cumulative evidence (e.g., publication bias, selective reporting within studies). | 5 |  |
| Additional analyses | 16 | Describe methods of additional analyses (e.g., sensitivity or subgroup analyses, meta-regression), if done, indicating which were pre-specified. | 5 |  |
| **RESULTS** | | |  |  |
| Study selection | 17 | Give numbers of studies screened, assessed for eligibility, and included in the review, with reasons for exclusions at each stage, ideally with a flow diagram. | 5-6 |  |
| Study characteristics | 18 | For each study, present characteristics for which data were extracted (e.g., study size, PICOS, follow-up period) and provide the citations. | 6 |  |
| Risk of bias within studies | 19 | Present data on risk of bias of each study and, if available, any outcome level assessment (see item 12). | 6 |  |
| Results of individual studies | 20 | For all outcomes considered (benefits or harms), present, for each study: (a) simple summary data for each intervention group (b) effect estimates and confidence intervals, ideally with a forest plot. | 6 |  |
| Synthesis of results | 21 | Present results of each meta-analysis done, including confidence intervals and measures of consistency. | 8 |  |
| Risk of bias across studies | 22 | Present results of any assessment of risk of bias across studies (see Item 15). | 8 |  |
| Additional analysis | 23 | Give results of additional analyses, if done (e.g., sensitivity or subgroup analyses, meta-regression [see Item 16]). | 8 |  |
| **DISCUSSION** | | |  |  |
| Summary of evidence | 24 | Summarize the main findings including the strength of evidence for each main outcome; consider their relevance to key groups (e.g., healthcare providers, users, and policy makers). | 8-9 |  |
| Limitations | 25 | Discuss limitations at study and outcome level (e.g., risk of bias), and at review-level (e.g., incomplete retrieval of identified research, reporting bias). | 9 |  |
| Conclusions | 26 | Provide a general interpretation of the results in the context of other evidence, and implications for future research. | 9 |  |
| **FUNDING** | | |  |  |
| Funding | 27 | Describe sources of funding for the systematic review and other support (e.g., supply of data); role of funders for the systematic review. | no |  |

*From:*  Moher D, Liberati A, Tetzlaff J, Altman DG, The PRISMA Group (2009). Preferred Reporting Items for Systematic Reviews and Meta-Analyses: The PRISMA Statement. PLoS Med 6(7): e1000097. doi:10.1371/journal.pmed1000097

For more information, visit: **www.prisma-statement.org**.

Page 2 of 2
